# Chromosomal instability (CIN) in HAP1 cell lines revealed by multiplex fluorescence in situ hybridisation (M-FISH)

**DOI:** 10.1186/s13039-022-00625-x

**Published:** 2022-10-26

**Authors:** Ruby Banerjee, Cibele G. Sotero-Caio, Beiyuan Fu, Fengtang Yang

**Affiliations:** 1 Molecular Cytogenetics Core Facility, Wellcome Sanger Institute, Cambridge, CB10 1SA UK; 2 School of Life Sciences and Medicine, Shandong University of Technology, Zibo, Shandong People’s Republic of China

**Keywords:** HAP1, Fluorescence in situ hybridisation, Chromosomal instability, Karyotype heterogeneity, CRISPR-Cas9 gene editing, Diploidisation

## Abstract

**Background:**

HAP1, a near-haploid human leukemic cancer cell line is often used in combination with CRISPR-Cas9 gene editing technology for genetic screens. HAP1 carries the Philadelphia chromosome (Ph) and an additional ~ 30 Mb fragment of chromosome 15 inserted into chromosome 19. The potential use of an in vitro cell line as a model system in biomedical research studies depends on its ability to maintain genome stability. Being a cancer cell line with a near-haploid genome, HAP1 is prone to genetic instability, which is further compounded by its tendency to diploidise in culture spontaneously. Moreover, CRISPR-Cas9 gene editing coupled with prolonged in-vitro cell culturing has the potential to induce unintended ‘off-target’ cytogenetic mutations.

To gain an insight into chromosomal instability (CIN) and karyotype heterogeneity, 19 HAP1 cell lines were cytogenetically characterised, 17 of which were near-haploids and two double-haploids, using multiplex fluorescence in situ hybridisation (M-FISH), at single cell resolution. We focused on novel numerical (N) and structural (S) CIN and discussed the potential causal factors for the observed instability. For each cell line we examined its ploidy, gene editing status and its length of in-vitro cell culturing.

**Results:**

Sixteen of the 19 cell lines had been gene edited with passage numbers ranging from 10 to 35. Diploidisation in 17 near-haploid cell lines ranged from 4 to 35% and percentage of N- and S-CIN in [1n] and [2n] metaphases ranged from 7 to 50% with two cell lines showing no CIN. Percentage of cells with CIN in the two double-haploid cell lines were 96% and 100% respectively. The most common S-CIN observed was deletion followed by translocation of both types, non-reciprocal and Robertsonian. Interestingly, we observed a prevalence of S-CIN associated with chromosome 13 in both near-and double-haploid cell lines, with a high incidence of Robertsonian translocation involving chromosome 13. Furthermore, locus-specific BAC (bacterial artificial chromosome) FISH enabled us to show for the first time that the additional chromosome 15 fragment is inserted into the p-arm rather than the q-arm of chromosome 19 of the HAP1 genome.

**Conclusion:**

Our study revealed a high incidence of CIN leading to karyotype heterogeneity in majority of the HAP1 cell lines with the number of chromosomal aberrations varying between cell lines. A noteworthy observation was the high frequency of structural chromosomal aberrations associated with chromosome 13. We showed that CRISPR-Cas9 gene editing technology in combination with spontaneous diploidisation and prolonged in-vitro cell culturing is potentially instrumental in inducing further chromosomal rearrangements in the HAP1 cell lines with existing CIN. We highlight the importance of maintaining cell lines at low passage and the need for regular monitoring to prevent implications in downstream applications. Our study also established that the additional fragment of chromosome 15 in the HAP1 genome is inserted into chromosome 19p rather than 19q.

**Supplementary Information:**

The online version contains supplementary material available at 10.1186/s13039-022-00625-x.

## Introduction

Chromosomal instability (CIN) is a form of genomic instability that encompasses ongoing changes in chromosome complements resulting in abnormal DNA content in cells. CIN involves changes in chromosome number (N-CIN), by gains or losses of whole chromosomes; or chromosome structure (S-CIN) by partial gains or losses of chromosomes such as deletions, translocations, amplifications, inversions and complex rearrangements. Both N- and S-CIN often co-exist within a given cell or tumour. Continuous acquisition of novel chromosomal aberrations generates cell to cell variations resulting in karyotype heterogeneity. [1, 2].

Assessment of CIN is important for model *in-vitro* cell lines used in biomedical research. Rigorous quality control of karyotype integrity is required in cell lines used in studies of gene expression and function, response to drugs and pathogens, elucidation of cellular mechanisms as well as of drug discovery. Ideally, the rates of CIN in model *in-vitro* cell lines should be low enough to ensure that significantly different phenotypes observed between ‘treated’ and ‘control’ experiments are the ‘effect’ of target treatments and not the result of differential expression of cells with clonal chromosomal alterations. Despite the importance of karyotype assessment, few studies have focused on reporting levels of CIN in currently used *in-vitro* model cell lines, particularly those that are inherently unstable or cancer derived like HAP1, as well as those bought commercially and kept at high passage across laboratories and institutions.

**Fig. 1:**
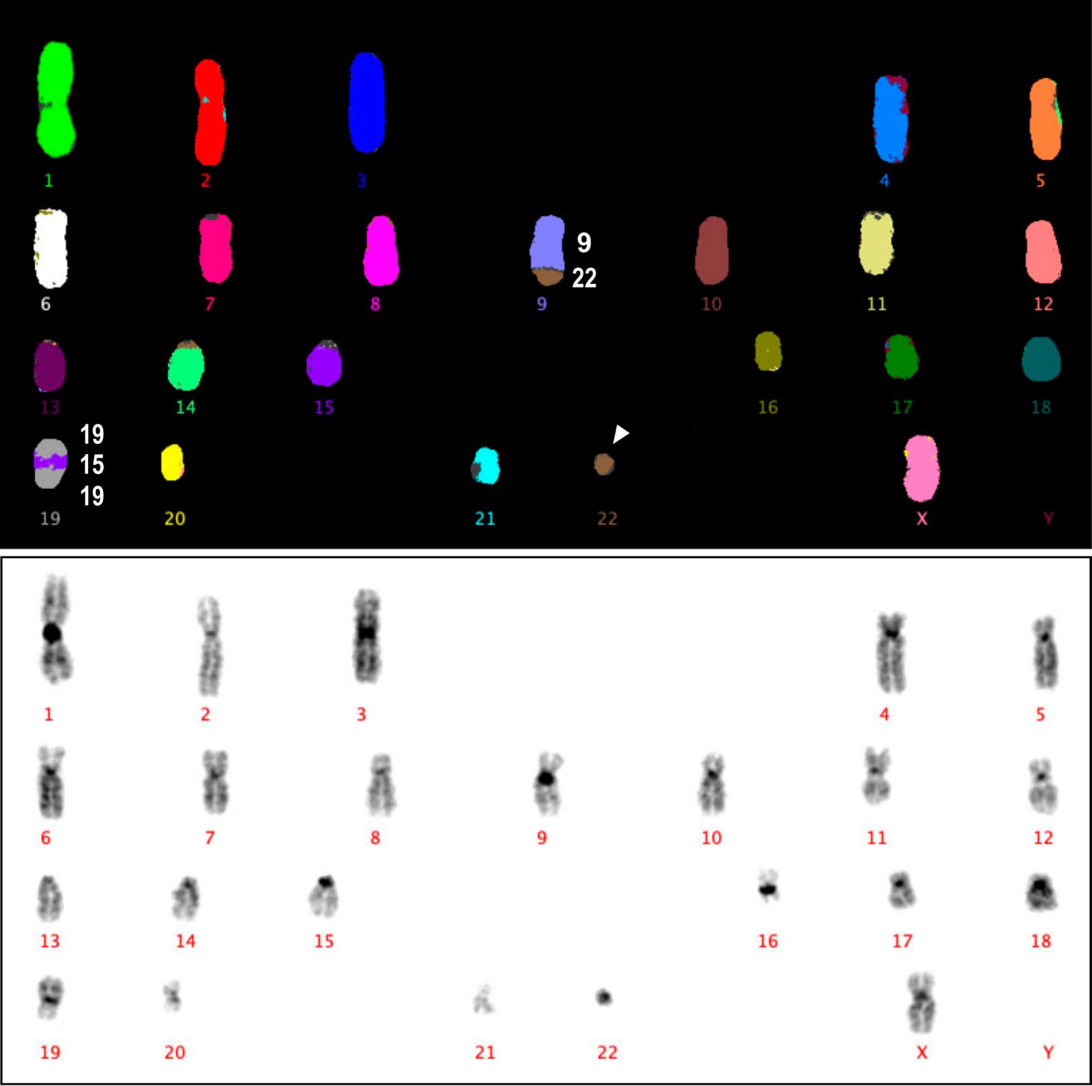
Karyotype of HAP1 metaphase. 23, X, t(9;22), ins(19;15); M-FISH and respective inverted DAPI band images show the constitutional chromosomal complement

In this context, the near-haploid HAP1 cell line, is a powerful model, widely used in gene function studies because mutation of a single allele causes loss-of-function phenotypes in the cell line. Of a male chronic myeloid leukemic origin, HAP1 is a fibroblast – like derivation of the near-haploid KBM-7 cell line [3, 4]. Between HAP1 and KBM-7 there are few karyotype differences, however HAP1 has gained the ability to grow as an adherent cell line and lack expression of hematopoietic markers. It has a haploid karyotype (Fig. 1) except for an additional fragment of chromosome 15, ~ 30 Mb long, inserted into chromosome 19 and constitutionally carries a reciprocal translocation of chromosomes 9 and 22, the Philadelphia chromosome (Ph), common in CML [5, 6]. Among the primary reasons favouring HAP1 as a model cell line in biomedical research laboratories worldwide, is its immense potential for gene editing by the CRISPR-Cas9 technology, a powerful technique used to invalidate genes for functional studies.

Despite its popularity in biomedical research, CIN in HAP1 cell lines remains under-reported and it is unknown to which extent new unintended chromosome alterations arise, whether some rearrangements are prone to occur more than others and if there is a correlation between the gene editing method (CRISPR-Cas9) and the appearance of novel chromosome aberrations.

The current information can be summarised as below:

1. HAP1 being a cancer cell line is inherently unstable; its near-haploid genome is prone to spontaneous ‘diploidisation’ due to a rapid enrichment of diploid cells in culture. To avoid this, such cell lines are frequently flow sorted to maintain a high percentage of haploid cells [7]
2. Accumulation of CIN is further enhanced by prolonged in-vitro cultures [5]
3. Gene editing technology, CRISPR-Cas9 can cause ‘off-target’ and sometimes, ‘at or near-target loci’ unintended mutations manifested as large-scale chromosomal rearrangements; thus, use of this technique comes with the increased need of monitoring cell line genome integrity [8, 9].

Therefore, to gain an insight into CIN we undertook a detailed analysis of characterisation of chromosomal aberrations in HAP1 cell lines with the help of M-FISH karyotyping, a single cell-based assay. M-FISH has an advantage over molecular based analyses which use pools of cells, it provides information about individual cells, identifying cryptic chromosomal rearrangements whilst reflecting inter- and intra-tumour genomic changes leading to karyotype heterogeneity in the cell lines.

In our study, we characterised CIN in 19 HAP1 cell lines majority of which had been CRISPR-Cas9 gene edited and underwent extended *in-vitro* cell culturing. We focussed on novel N- and S-CIN and explored the potential underlying causal factors that may have triggered CIN in the cell lines.

Further, FISH with locus-specific probes derived from bacterial artificial chromosome (BAC) clones provided information on the correct localisation and orientation of the additional ~ 30 Mb long chromosome 15 fragment into the p-arm of chromosome 19 of the HAP1 genome.

Overall, our study revealed CIN and the extent of it in the HAP1 cell lines with variations in the rate of CIN between cell lines. We highlight the importance of regular monitoring of unstable cell lines for genetic instability due to novel, unintended and undetected genomic alterations to avoid further implications in downstream analyses leading to misinterpretation of data.

## Results

### Localisation of the additional chromosome 15 fragment into chromosome 19p of the HAP1 genome using locus-specific BAC FISH

Using metaphases from the reference cell line GM15510 and HAP1 cell line 4C1-R1 for locus-specific FISH we were able to validate the localisation and orientation of the probes selected to map to regions of chromosomes 15 and 19.

**Fig. 2:**
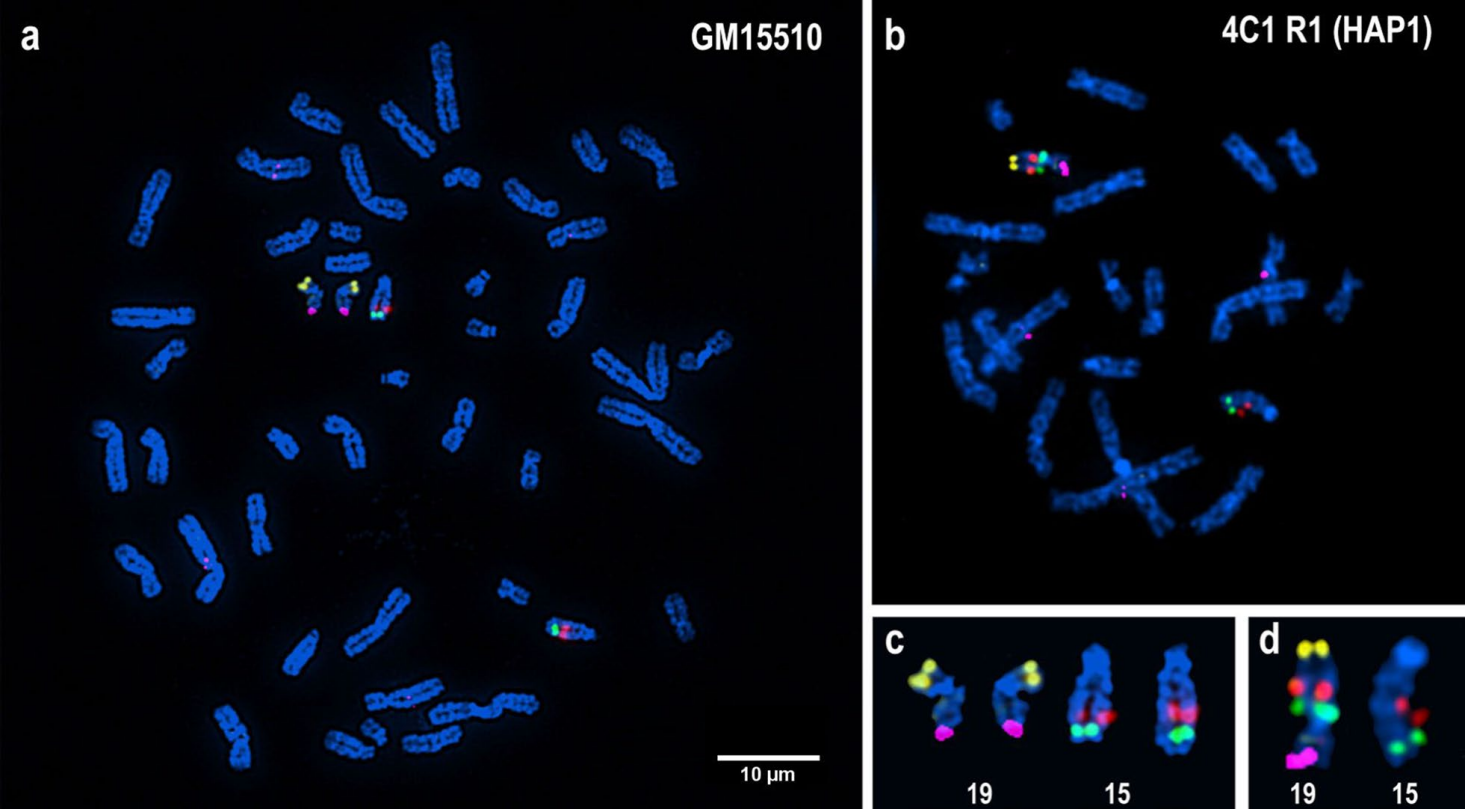
Locus-specific BAC FISH localization of the additional chromosome 15 fragment into chromosome 19p. Images represent hybridization results of locus-specific FISH on metaphase chromosomes of **a** GM15510, control cell line and **b** 4C1 R1, HAP1 cell line. Individual chromosomes from images **a** and **b** have been highlighted in images **c** and **d** respectively. Image c shows RP11-537K8 (15q22.31; red) and RP11-43K17 (15q25.3; green) mapping to chromosome 15; RP11-333F10 (19p13.3; yellow) and RP11-45N1 (19q13.43; pink) mapping to chromosome 19 in control cell line GM15510. Image d shows RP11-537K8 (15q22.31; red) and RP11-43K17 (15q25.3; green) mapping to chromosome 19p of HAP1 cell line 4C1 R1 demonstrating that the additional fragment of chromosome 15 is inserted and oriented in an inverted pattern onto the ‘p’ arm of chromosome 19

FISH with BAC probes (Fig. 2, Table 1 and Method 1) demonstrated that the additional ~ 30 Mb (Chr. 15:61,105,000 to Chr. 15:89,890,000) fragment of chromosome 15, encompassing almost 30 million base pairs, is inserted into the ‘p’ arm of chromosome 19 and not the ‘q’ arm (long) of the chromosome (Fig. 2) as reported by Esseltzbichler et al. [6].

**Table 1:** BAC probes for locus-specific FISH

BAC	Fluorescent dUTP
RP11-537K8 (15q22.31)	Texas Red dUTP (Jena bioscience)
RP11-43K17 (15q25.3)	Atto 488 XX dUTP (Jena bioscience)
RP11-333F10 (19p13.3)	Cy3 dUTP (Jena bioscience)
RP11-45N1 (19q13.43)	Cy5 dUTP ( Jena bioscience)

BAC probes for specific-locus FISH, labelled directly with Texas Red-dUTP, Atto-488-XX-dUTP, Cy3-XX-dUTP and Cy5-XX-dUTP respectively, for metaphase FISH

### Characterisation of HAP1 cell lines by M-FISH

M-FISH karyotyping and DAPI banding allowed us to investigate N-and S-CIN and the extent of karyotype heterogeneity in 19 HAP1 cell lines, of which 17 were near-haploids and two double-haploids. We focused on novel chromosomal rearrangements while examining the underlying causal factors that may have triggered CIN in the cell lines. For each cell line we examined ploidy, CRISPR-Cas9 gene editing status and the length of *in-vitro* cell culturing as outlined in Table 2.

**Table 2:** HAP1 cell lines analysed in the study

Cell line ID and ploidy status	CRISPR-Cas9 gene editing	Number of passages in cell culture	Percentage of haploidy based on 100 metaphases (%)	Percentage of diploidy based on 100 metaphases (%)
HAP1 Pool Lig4-Cas9- [1n]	Yes	~ 20	88	12
C6 HAP1 [1n]	Yes	~ 30	89	11
B3 HAP1 P17 [1n]	Yes	~ 33	85	15
1 N-HAP1-P19 [1n]	Yes	~ 35	85	15
HAP1 A1 [1n]	Yes	~ 30	96	4
HAP1 A2 [1n]	Yes	~ 30	88	2
HAP1 A5 [1n]	Yes	~ 30	92	8
HAP1 E5 [1n]	Yes	~ 30	81	19
HAP1 F3 [1n]*	Yes	~ 30	90	10
HAP1 G2 [1n]	Yes	~ 30	78	22
HAP1-HO-C [1n]	No	~ 15	92	8
HAP1 GR 5.10 [1n]	No	Unknown	92	8
HAP1 GR S2 [1n]	No	Unknown	96	4
4C1 R1 [1n]	Yes; LIG4 KO	Minimum 10	85	15
4C1 R2 [1n]	Yes; LIG4 KO	Minimum 10	93	7
19C1 R1 [1n]	Yes; LIG4 & p53 KO	MINIMUM 10	85	15
HAP1 Pool CAS9 + BLAST [1n]	Yes	~ 30	65	35
HAP1-2 N-C [2n]	Yes	~ 15	–	100
HAP1-P53-KO [2n]	Yes	~ 20	–	100

Sixteen of the 19 HAP1 cell lines underwent gene editing by CRISPR-Cas9 technology. *In-vitro* cell culturing or ‘passage numbers’ of the cell lines ranged from a minimum of 10 (4C1 R1, 4C1 R2 and 19C1 R1) to a maximum of 35 (1 N HAP1-P19) with unknown passage numbers in two cell lines namely HAP1 GR 5.10 and HAP1 GR S2. N- and S-CIN resulting in karyotype heterogeneity was observed in 15 near-haploid and two double-haploid cell lines. Number of novel chromosomal aberrations varied between cell lines as enlisted in Additional file 1: Tables S1 (near-haploid cell lines) and Additional file 2: Table S2 (double-haploid cell lines).

HAP1 cell lines showing ploidy status, gene editing status, passage numbers of cell culture and percentages of haploidy and diploidy. *HAP1 F3 carries 1 triploid metaphase [3n].

### CIN in near-haploid cell lines

Diploidisation, a spontaneous phenomenon of haploid metaphases in near-haploid [1n] cell lines becoming double-haploid [2n] metaphases over time, ranged from 4 (HAP1 A1 and HAP1 GR S2) to 35% (HAP1 Pool Cas9 + Blast) (Table 2).

Percentage of CIN, in [1n] and [2n] metaphases ranged from 7% (HAP1 Pool Lig4-Cas9-) to 50% (HAP1 Pool Cas9 + Blast). No CIN was observed in two cell lines viz. C6 HAP1 and HAP1 GR S2. In [1n] metaphases percentage of CIN ranged from 3 to 40% and prevalence of S-CIN was observed over N-CIN. Percentage of CIN in [2n] metaphases ranged from 3 to 30% and both N- and S-CIN were observed in the metaphases of the cell lines. It must be noted that often both N- and S-CIN co-existed in the same metaphase and that the majority of the cell lines with high passage numbers showed CIN with the exception of C6 HAP1 (Table 3 and Additional file 1: Table S1).

**Table 3:** Percentage of CIN in near-haploid HAP1 cell lines

Near haploid cell line ID and number of metaphases karyotyped	Percentage of N- and S-CIN in [1n] metaphases (%)	Percentage of N- and S-CIN in [2n] metaphases (%)	Total number of N- and S-CIN in [1n] and [2n] metaphases	Number of passages in cell culture	CRISPR-Cas9 gene editing
HAP1 Pool Lig4-Cas9[30]	None	7	7%[2]*	~ 20	Yes
B3 HAP1 P17[30]	20	7	27%[8]*	~ 33	Yes
1 N HAP1 P19[30]	7	3	10%[6]*	~ 35	Yes
HAP1 A1[30]	17	3	20%[6]*	~ 30	Yes
HAP1 A2[30]	10	7	17%[5]*	~ 30	Yes
HAP1 A5[30]	10	3	13%[4]*	~ 30	Yes
HAP1 E5[30]	23	10	33%[10]*	~ 30	Yes
HAP1 F3[30]	17	13	30%[9]*	~ 30	Yes
HAP1 G2[30]	20	7	27%[8]*	~ 30	Yes
HAP1-HO-C[25]	16	None	16%[4]*	~ 15	Yes
HAP1 GR 5.10[25]	40	None	40%[10]*	Unknown	No
4C1 R1[50]	6	12	18%[9]*	Minimum 10	No
4C1 R2[50]	4	12	16%[8]*	Minimum 10	No
19C1 R1[50]	6	12	18%[9]*	Minimum 10	Yes; LIG4 KO
HAP1 Pool Cas9 + Blast[30]	20	30	50%[15]*	~ 30	Yes; LIG4 KO
C6 HAP1[30]	–	–	None	~ 30	Yes; LIG4 & p53 KO
HAP1 GR S2[25]	–	–	None	Unknown	Yes

Percentage of N- & S-CIN in near-haploid cell lines and passage numbers

*Number of metaphases with N- & S-CIN (n.b. percentages include chromosome break)

**Fig. 3:**
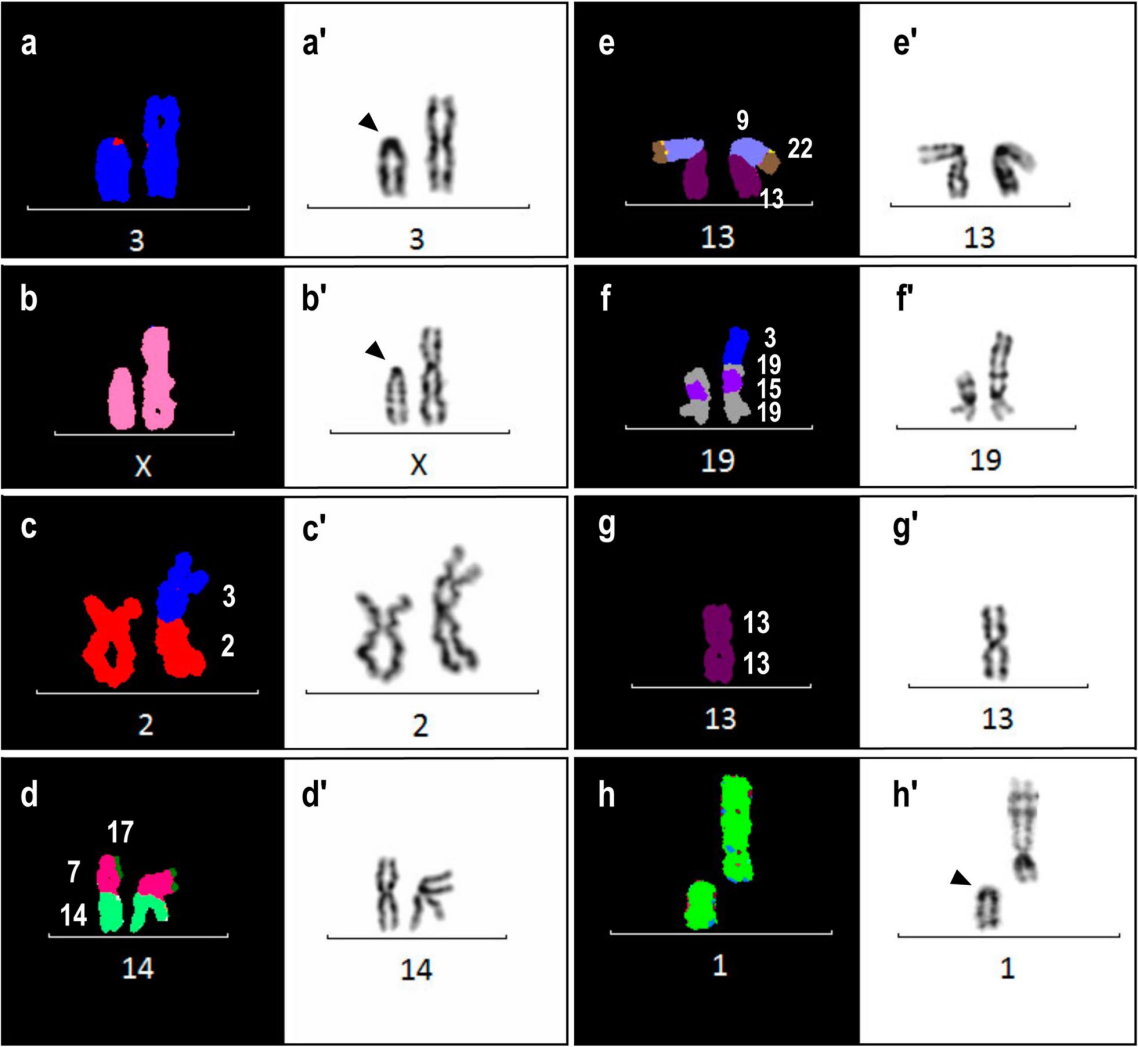
Examples of structural rearrangements observed in HAP1 cell lines. M-FISH (left) and respective inverted DAPI-banding (right) of individual chromosomes from multiple metaphases of HAP1 cell lines analysed in the present study. Chromosome identification is shown under each chromosome (or homologues). Deletions (**a**, **b**), non-reciprocal translocations (**c**, **d**), complex rearrangements (**e**, **f**), Robertsonian translocation (**g**), and chromosome break (**h**) are shown. Black arrowhead points to site of chromosome breakage

The most common S-CIN identified were deletions followed by translocations of both types, non-reciprocal and Robertsonian. More segmental gains of chromosomes were observed in [1n] metaphases in comparison to [2n] metaphases where more segmental losses were observed instead. Chromatid and chromosome breaks (chtb and chrb) have also been observed in the cell lines. Incidentally, twelve cell lines carried deletions and the percentage ranged from 3 (HAP1 A2) to 23% (B3 HAP1 P17). Robertsonian translocation, yet another prevalent structural aberration was observed in 8 near-haploid cell lines (47%), majority of which were associated with chromosome 13. Figure 3 shows examples of different types of S-CIN observed in the HAP1 cell lines.

Novel rearrangements identified may indicate potential clonality as observed in certain cell lines such as HAP1 GR 5.10 with three metaphases carrying a derivative involving chromosomes 9 and 13 [der(13)t(9;13)], (Additional file 1: Table S1 and Fig. 5). This particular derivative has also been observed in three other near-haploid cell lines (S1). HAP1 Pool Cas9 + Blast and HAP1 GR 5.10 show extensive karyotype heterogeneity with percentage of N- and S-CIN being 50% and 40% respectively (S1). Here, it must be pointed out that some of the numerical aberrations could be a technical artefact due to overspreading of metaphases as observed in HAP1 Pool Cas9 + Blast.

**Fig. 4:**
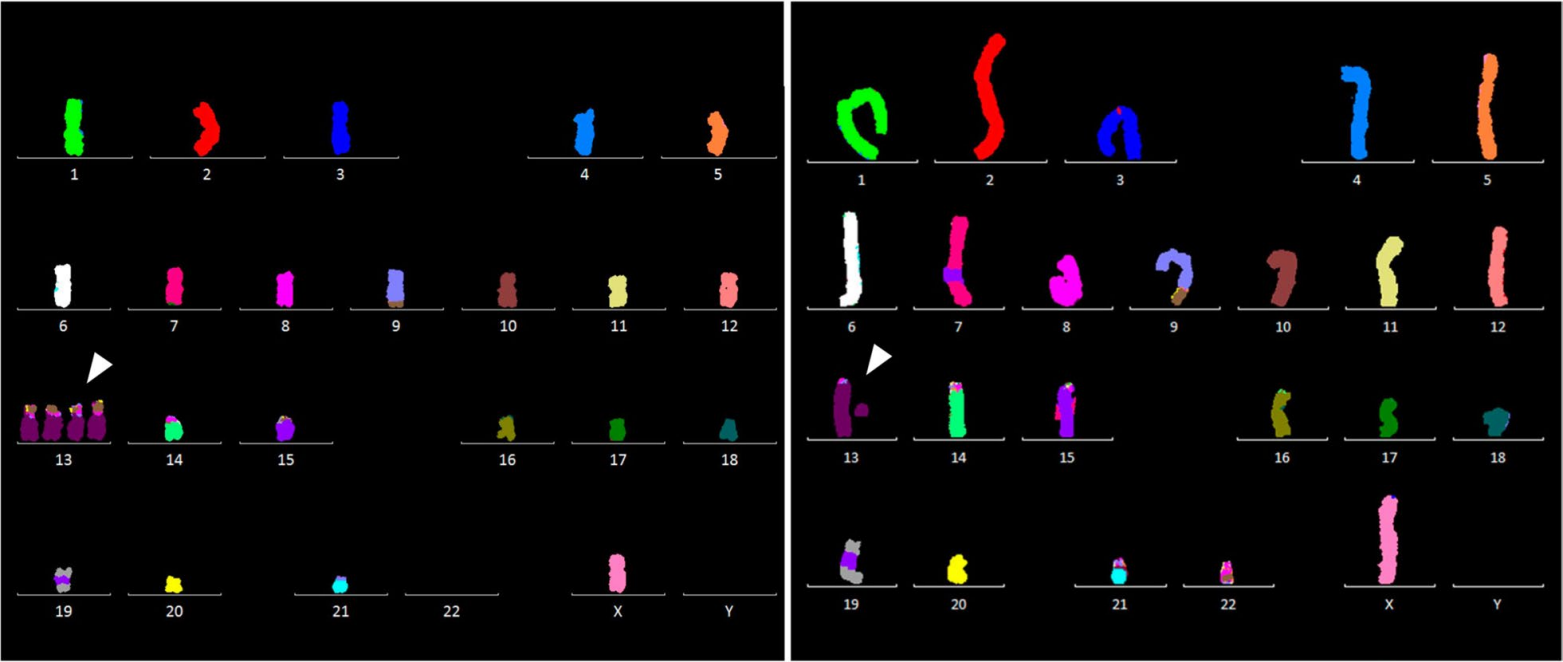
Metaphases illustrating aneuploidy of chromosome 13 in HAP1 cells. Karyotypes of metaphases displaying whole chromosome (left) or partial (right) gain of chromosome 13. White arrowheads highlight the positions of chromosome 13 in the karyotype

**Fig. 5:**
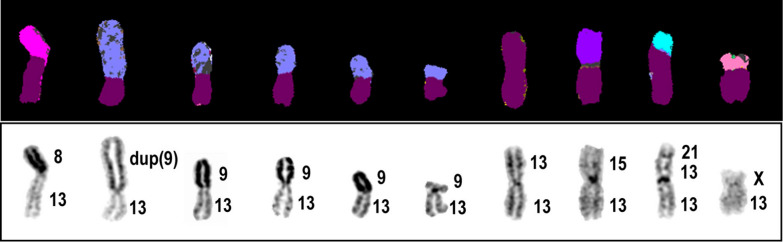
S-CIN involving chromosome 13 in 10 metaphases of HAP1 GR 5.10. Individual copies of chromosome 13 from multiple metaphases are displayed in a row in this composite image. The top row corresponds to M-FISH and bottom row shows their respective inverted DAPI band images, with chromosome notation to the right side of each chromosome

Interestingly, the highest frequency of CIN in the near-haploid cell lines has been associated with chromosome 13. As enlisted in Table 4, twelve cell lines out of 17 (~ 71%) have shown CIN involving chromosome 13 revealing partial and whole copy gains and losses. Segmental and whole copy chromosomal gains of chromosome 13 have been observed in [1n] metaphases as illustrated in Figs. 4 and 5. Novel rearrangements involving the chromosome were more structural than numerical. Eleven cell lines showed mainly S-CIN which were non-reciprocal translocations, Robertsonian translocations and deletions in both [1n] and [2n] metaphases. Robertsonian translocations were observed in 8 (47%) near-haploid cell lines where the translocations involved two copies of chromosome 13 mainly. Robertsonian translocations between chromosome 13 and other acrocentric chromosomes (14 and 15) have also been observed. Of particular note are 10 [1n] metaphases in near-haploid cell line HAP1 GR 5.10 which carried S-CIN associated with chromosome 13 (Table 4 and Fig. 5).

**Table 4:** CIN associated with chromosome 13 in near-haploid HAP1 cell lines

HAP1 Cell line ID	Non-reciprocal translocation involving chromosome 13 [1n & 2n]		Robertsonian translocation involving chromosome 13[1n & 2n]		Deletion involving chromosome 13[1n & 2n]		N-CIN involving chromosome 13[1n & 2n]	
	[1n]	[2n]	[1n]	[2n]	[1n]	[2n]	[1n]	[2n]
HAP1 Pool Lig4- Cas9-								− 13[2n]
B3 HAP1 P17				rob(13;13)[2n]				
	der(13)t(9;13)[1n]							
HAP1 A1					+ del(13)[1n]			
HAP1 E5	der[13;(22)t(9;22)], + der[13;(22)t(9;22)] × 3[1n]							
					+del(13)[1n]			
							+13[1n]	
				rob(13;13)[2n]				
HAP1 F3				rob(13;13)[2n]				
								− 13[2n]
								+13 × 3[3n]
HAP1 G2	der(13)t[der(22)][1n]							
HAP1-HO-C	der(13)t(3;13)[1n]							
	der(13)t(13;20)[1n]							
	der(13)t(5;13)[1n]							
HAP1 GR 5.10	der(13)t(8;13)[1n]							
	der(13)t(9;13)[1n][4]*							
	der(13)t[dup(9);13][1n]							
			rob(13;13)[1n]					
			rob(13;15)[1n]					
			rob(13;21)[1n]					
	der(13)t(X;13)[1n]							
4C1 R1				rob(13;13)[2n]				
				rob(13;13)[2n]				
4C1 R2		der(13)t(13;15;22) × 2[2n]						
				rob(13;13)[2n]				
19C1 R1				rob(13;15)[2n]				
				rob(13;14) × 2[2n]				
						del(13)[2n]		
HAP1 Pool Cas9+Blast	der(13)t(9;13)							
				rob(13.13)[2n]				
								− 13[2n]

Novel N- and S-CIN associated with chromosome 13 in near-haploid HAP1 cell lines; * indicates number of metaphases

The two double haploid cell lines displayed extensive karyotype heterogeneity with chromosomal changes that had occurred in the near-haploid metaphases from which they had originated (see Additional file 2: Table S2).

HAP1-2n-C and HAP1-P53-KO show 96% and 100% CIN respectively. S-CIN observed in the two cell lines were similar to what was observed in the near-haploid cell lines. More S-CIN was observed in HAP1-2n-C unlike HAP1-P53-KO which showed both N- and S-CIN. Rearrangements involving chromosome 13, including non-reciprocal and Robertsonian translocations, have been observed in both cell lines. There are indications of potential clonality in both cell lines. For example, loss of a copy of one chromosome X (-X) was observed in six out of 30 metaphases in HAP1-P53-KO along with del(Xp) in five metaphases, as enlisted in S2. Similarly in HAP1-2n-C, a diploid cell line, with del(Xp) has been observed in five out of 25 metaphases.

## Discussion

Identifying the precise location and orientation of a sequence within a genome is important to define the linkage background for genes on a chromosome and is relevant for downstream applications, especially to determine whether position and orientation changes impact gene expression. We have demonstrated for the first time that the ~ 30 Mb additional fragment of chromosome 15 is inserted in an inverted orientation onto the ‘p’ arm of chromosome 19 and not the ‘q’ arm as reported previously by Esseltzbichler et. al. [6].

Haploid genetics has always been important for understanding genome evolution and function. With natural haploidy absent in Vertebrates, scientists have often attempted to obtain haploid cells from fish, mice and particularly embryonic stem cells via culturing methods [10]. However, generation and maintenance of haploid model lines has been challenging despite the use of human tumour cells where chromosome numbers within cells are severely reduced often resulting in hypodiploid or near-haploid tumours [3, 10–13]. KBM-7, a near-haploid cell line originating from a male with chronic myelogenous leukemia [4] is the progenitor of the HAP 1 cell line, and both have revolutionised biomedical research by becoming the most important near-haploid *in-vitro* model often used in gene editing [14, 15]. However, the usefulness of cancer cell lines as *in-vitro* models particularly in therapeutic applications is highly dependent on their genome integrity and reproducibility of data. This is true for haploid models, such as HAP-1, where maintenance of a haploid state throughout experiments is crucial to the testing of hypotheses. Hence, genome stability in this cell line is crucial for any downstream analyses and applications.

In this study, we speculated that the underlying instability of the HAP1 cell line due to its inherent haploid status coupled with CRISPR-Cas9 machinery may have been potentially instrumental in inducing CIN in the form of ‘unintended off-target’ chromosomal aberrations [2, 8]. Accumulation of CIN may have been further increased by the cell lines spontaneous ‘diploidisation’ property together with prolonged cell culturing.

One common feature of the cell lines analysed in the study was the presence of diploid cells in an otherwise haploid model. Ploidy instability, leading to diploidisation is a natural feature of haploid cultures in general. For instance, decrease in the percentage of haploid cells has been observed by several authors [7, 16], in human cells. Olbirch et al. (2017) showed that loss of haploidy is due to an overgrowth of diploid cells present in the cultures since haploid cells are less viable due to activation of a p53-dependent response. Beigl et al. [5] observed that HAP1 cell cultures became diploid within a short time-frame, approximately around 20 passages, post CRISPR-Cas 9 gene editing, and this could increase with more passages. In our study, as previously mentioned, the near-haploid cell lines, with passage numbers ranging from a minimum of ~ 10 to a maximum of ~ 35 showed diploidy percentage range of 2 to 35%, including 2 cell lines (HAP1 GR 5.10 and HAP1 GR S2) with unknown passage numbers (Table 2).

During diploidisation, HAP1 cells in culture may potentially develop CIN generating intra-lineage diversity due to progressive accumulation of new chromosomal aberrations, thus forming heterogeneous karyotypes within a cell population. CIN increases further in such unstable cell lines by prolonged *in-vitro* cell culturing and a growing number of scientific publications demonstrate that passage number affects a cell line’s characteristic over time. Cell lines at high passage numbers experience alterations often manifested as chromosomal aberrations resulting in cell to cell heterogeneity, eventually heterogeneous karyotypes, in comparison to lower passaged cell cultures. The evidence for passage number related effects on cell lines is compelling [8, 17]. Thus, cell line quality is crucial to any experiment and avoiding cell lines that have been in culture for too long is an important step to ensure reliable and reproducible results [8, 18].

Seventeen out of 19 HAP1 cell lines, in our study, had been CRISPR-Cas9 gene edited. HAP1, an unstable cancer cell line when gene edited by CRISPR-Cas9 may have an increased level of genome instability in comparison to primary cell lines with stable karyotypes. Incidentally, primary cell lines had been used in the development of CRISPR methodology [8]. As previously mentioned, the disadvantage of using this gene editing technique is that it may induce unintended mutations ‘off-target’ and ‘at or within target loci’ often manifested as chromosomal aberrations as observed by Rayner et. al. [8]. This observation has been supported in a previous study by Alanis-Lobato et. al. (2021)[9] where the authors draw attention to unintended consequences of the technique to gene edited human germ lines. In addition, large insertions and deletions at or near target loci have also been reported in gene edited mice, mouse embryonic stem cell and human differentiated cells. [3]. Thus, the effects of CRISPR-Cas9 induced chromosomal mutations in cell lines like the HAP1, not yet fully investigated, underscores the importance of checking and controlling such unintended chromosomal aberrations. The mutations may remain undetected even after using screening methods like PCRs and/or long read sequencing technologies. Whole genome sequencing, though analytically powerful, is expensive and not available universally. Off-target mutations would bring about differences between parental and mutated cell lines and affect downstream analyses leading to flawed or misinterpretation of results.

In our study S-CIN was more prevalent than N-CIN. The three main structural aberrations observed were deletions, non-reciprocal translocations and Robertsonian translocations. But the predominant structural CIN observed was deletion. Chromosomal deletion has always been described as one of the hallmarks of cancer and deleted regions have been widely demonstrated to contain tumour suppressor genes. Such segmental losses from chromosomes provide the cells with selective growth advantage particularly in unstable cancer-derived cell lines like HAP1 [19, 20].

Chromosomal translocation is yet another hallmark of cancer that drives genome instability [21, 22]. Non-reciprocal translocations have been widely observed in the cell lines of our study. Like deletions non-reciprocal translocations may also give rise to segmental gains and losses of chromosomes bearing oncogenes or tumour suppressor genes. Another prevalent S-CIN observed in this study was Robertsonian translocation often associated with chromosome 13. Considered one of the most common chromosomal rearrangements observed in human cells Robertsonian translocations involve the fusion of two acrocentric chromosomes. This translocation is often observed in haematological disorders with predisposition to malignancies. [23, 24].

Our study has shown a high frequency of chromosomal aberrations associated with chromosome 13 (Table 4). Eleven near-haploid cell lines, approximately 65%, showed S-CIN associated with the chromosome in the form of deletions, non-reciprocal translocations and predominantly Robertsonian translocations. S-CIN was more prevalent than N-CIN. In [1n] metaphases of the cell lines whole copy chromosomal losses were not observed since such karyotypic changes in metaphases are not compatible with cell viability. Chromosome 13 is the largest acrocentric human chromosome, characterised by a certain level of plasticity that has been implicated in many human cancers and diseases [25, 26]. Forty-eight mendelian conditions listed in ‘Online Mendelian inheritance in Man’ (OMIM) have been linked to genes on chromosome 13. *BRCA2* gene, retinoblastoma gene and the alveolar rhabdomyosarcoma gene *FOXO1A* to mention a few, have been identified on chromosome 13. B cell chronic lymphocytic leukaemia (CLL) is one of the most common leukaemia in the western world and approximately 10% of CLL patients have a homozygous deletion in 13q14.3 [25]. Our observation of chromosome 13 structural aberrations in majority of the near-haploid cell lines perhaps reflects its chronic myelogenous leukemic (CML) origin.

M-FISH the technique used to analyse the cell lines is a cost-effective method for visualising chromosomal aberrations. As in any technique M-FISH has its limitation, it fails to detect S-CIN below 3–5 Mb. It is also labour intensive, requires live cells, assesses small sample sizes and is highly specialized. A more detailed comprehensive analysis of CIN could perhaps be provided by single-cell genomics a technique that is continually evolving with increasing accuracy of DNA amplification alongside novel methods that do not require pre-amplification of DNA [27]. However, while laboratories might not have the infrastructure for more complex routine methodologies, we highly recommend that cells in culture are routinely checked for new aberrations with M-FISH, especially after gene editing and after regular number of passages. [28, 29].

## Conclusion

In this study we have performed an in-depth molecular cytogenetic characterisation of 19 HAP1 cell lines by M-FISH karyotyping. M-FISH provided estimates of incidence of CIN in HAP1 lineages along with insights into possible events responsible for it. We conjectured that standard CRISPR-Cas9 gene editing technology in combination with diploidisation in an unstable near-haploid cancer cell line like HAP1 with existing CIN and undergoing prolonged in vitro cell culturing may have been instrumental in inducing further genome instability. Such chromosomal instability caused cell to cell variation resulting in karyotype heterogeneity, the rate of which varied between cell lines.

Locus-specific FISH refined the location and orientation of the additional ~ 30 Mb fragment of chromosome 15 showing its integration, in an inverted pattern, into the ‘p’ arm of chromosome 19 and not the ‘q’ arm of chromosome 19, as previously thought.

Our data brings further evidence to highlight the importance of maintaining cell lines used in biomedical research laboratories at low passage and the need for regular monitoring to avoid significant accumulation of mutations which may otherwise have important implications in basic research and clinical applications. We addressed the concern of genome instability in the HAP1 cell lines whilst exploring and conjecturing the potential underlying causal factors that may have induced instability in the cell lines.

## Method 1 (M1). Locus specific FISH to map the insertion of an additional fragment of chromosome 15 integrated into chromosome 19p

Metaphase suspensions of HAP1 cell line, 4C1 R1 and control cell line GM15510, a human female transformed cell line were used in the locus-specific FISH experiment. Metaphase chromosomes were harvested following a standard protocol [30]. The adherent cell line was treated with colcemid (Karyomax™ Colcemid™ solution in PBS, 10 μg/ml) to a final concentration of 0.1 μg/ml for 1.5 h. TrypLE Express enzyme 1 $\times$ (Thermofisher Scientific) was used to dissociate adherent cells to obtain a single cell suspension which was treated with hypotonic buffer (0.56% KCl in distilled water) for 12–14 min and subsequently fixed with Carnoy’s fixative, 3:1 (v/v) methanol:acetic acid (VWR). The cell suspension was used to make metaphase slides for FISH.

Human bacterial artificial chromosome (BAC) clones (Table 1) mapping to chromosome 15 within the region Chr. 15:61,105,000 to Chr. 15:89,890,000 and from the ‘p’ and ‘q’ arms of chromosome 19, respectively, were supplied by the clone archive team at the Wellcome Sanger Institute.

Probes were generated from purified BAC DNA by whole genome amplification using GenomePlex® Whole Genome Amplification kit (Sigma-Aldrich), as described previously by Gribble et. al [30]. Probes were directly labelled with fluorophore dUTPs (Table 1).

Metaphase slides made from the single cell suspensions were pre-treated for 10 min. in Acetone (Sigma-Aldrich) at room temperature followed by baking in a 62 °C oven for an hour. Slides were then denatured in an alkaline denaturation solution (0.5 M NaOH, 1.0 M NaCl, Sigma-Aldrich) for 7 to 8 min. followed by rinses in 1 M Tris–HCl (pH 7.4) solution and 1 $\times$ PBS, 4 min. each. Finally, slides were dehydrated through an ethanol series (70%, 90%, 100%) and air dried.

The remaining FISH procedures largely followed Gribble et. al [30]. The probe mix containing the four labelled BACs, human Cot-1 DNA was precipitated down in ethanol, then resuspended in hybridisation buffer and denatured at 65 °C for 10 min. After denaturation, an aliquot of probe mix (~ 10 μl) was added to each denatured slide. The hybridisation area covered with a 22 mm $\times$ 22 mm coverslip and sealed with Fixogum rubber cement, was then incubated at 37 °C in an incubator, overnight.

Post-hybridisation washes involved a 30 min wash in 2 $\times$ SSC at 37 °C for the removal of coverslips with a subsequent 5 min. stringent wash in 0.5 $\times$ SSC at 73 °C, followed by 5 min washes in 2 $\times$ SSC containing 0.05% Tween*20 (VWR) and 1 $\times$ PBS, at room temperature.

Slides were mounted in Vectashield® Vibrance™ Antifade mounting medium containing DAPI (4’, 6-diamidino -2- phenylindole), Vector laboratories.

Imaging was carried out using 63 $\times$ objective in a Zeiss Axiolmager D1 fluorescent microscope equipped with a Hamamatsu CCD camera and narrow bandpass filters for DAPI, Cy3, Cy5, Texas Red (Cy3.5) and FITC fluorescence. Metaphase images were captured and processed using the SmartCapture software (Digital Scientific, UK). 10 metaphases from each cell line were analysed. [31].

## Method 2 (M2) M-FISH characterisation of 17 near-haploid and 2 double- haploid cell lines

Metaphase chromosomes were harvested from the HAP1 cell lines (Table 5) following a standard protocol. The adherent cell lines were treated with colcemid (Karyomax™ Colcemid™ solution in PBS, 10 μg/ml) to a final concentration of 0.1 μg/ml for 1.5 h. TrypLE Express enzyme 1 $\times$ (Thermofisher Scientific) was used to dissociate adherent cells to obtain a single cell suspension which was treated with hypotonic buffer (0.56% KCl in distilled water) for 12–14 min and subsequently fixed with Carnoy’s fixative, 3:1 (v/v) methanol:acetic acid (VWR). The cell suspension was used to make metaphase slides for FISH followed by fixing slides in acetone (Sigma Aldrich) for 10 min. before baking at 62 °C for 30 min. Metaphase spreads were denatured in an alkaline denaturation solution (0.5 M NaOH,1.0 M NaCl) for 7 ½—8 min. This was followed by two subsequent washes in 1 M Tris–HCl (pH 7.4) and 1 $\times$ PBS, 4 min each. Slides were then dehydrated in a 70%, 90% and 100% ethanol series and air dried.

**Table 5:** HAP1 cell lines used in the study

**Near-haploid cell lines**
HAP1 Pool Lig4-Cas9-
HAP1 A1
HAP1 F3
HAP1 GRS2
HAP1 Pool Cas9+Blast
C6 HAP1
HAP1 A2
HAP1 G2
4C1 R1
B3 HAP1 P17
HAP1 A5
HAP1-HO-C
4C1 R2
1 N-HAP1-P19
HAP1 E5
HAP1 GR 5.10
19C1 R1
**Double-haploid cell lines**
HAP1-2n-C
HAP1-P53-KO

List of near-haploid and double haploid HAP1 cell lines used in the study

The probe mix of 24 colour human M-FISH paint was denatured at 65 °C for 10 min. before applying onto the denatured slides. Hybridisation was carried out over two nights at 37 °C. Post-hybridisation washes involved a 30 min wash in 2 $\times$ SSC at 37 °C for the removal of coverslips with a subsequent 5 min. stringent wash in 0.5 $\times$ SSC at 75 °C, followed by 5 min washes in 2 $\times$ SSC containing 0.05% Tween*20 (VWR) and 1 $\times$ PBS, at room temperature. Slides were mounted in Vectashield® Vibrance™ Antifade mounting medium containing DAPI (4’, 6-diamidino -2- phenylindole), Vector laboratories.

Imaging was carried out using 63 $\times$ objective in a Zeiss Axiolmager D1 fluorescent microscope equipped with a Hamamatsu CCD camera and narrow bandpass filters for DAPI, DEAC, FITC, CY3, Texas Red (Cy3.5) and Cy5 fluorescence. A minimum of 25 metaphases per sample were imaged (Table 3) by using the SmartCapture software (Digital Scientific, UK) followed by karyotyping using the SmartType Karyotyper (Digital Scientific, UK). [31].

## Supplementary Information

**Additional file 1: Table S1**. Novel N- and S-CIN in near-haploid cell lines.

**Additional file 2: Table S2**. Novel N- and S-CIN in double-haploid cell lines.

## Data Availability

This declaration is not applicable.
