# Supplementary material for: Chromosomal instability (CIN) in HAP1 cell lines revealed by multiplex fluorescence in situ hybridisation (M-FISH)
Source: Mol Cytogenet. 2022 Oct 26;15:46. doi: 10.1186/s13039-022-00625-x (PMC9609465; doi:10.1186/s13039-022-00625-x)
Supplement: Supplementary file 1 — Additional file 1: Table S1. Novel N- and S-CIN in near-haploid cell lines. [file 13039_2022_625_MOESM1_ESM.docx]

**Supplementary table 1 (S1)** CIN in near-haploid cell lines

| **Near-haploid cell line ID and number of metaphases karyotyped** | **Number of metaphases [1n & 2n] with novel structural rearrangements (S-CIN)** | **Number of metaphases [1n & 2n] with novel numerical rearrangements (N-CIN)** |
| --- | --- | --- |
|  |  |  |
| **HAP1 Pool Lig4-Cas9-[30]** | - | -13[2n] |
|  | - | -22[2n] |
|  | normal karyotype[1n] & [2n][28]* |  |
|  |  |  |
| **B3 HAP1 P17[30]** | chtb(14)[1n] |  |
|  | chrb(X)[1n] |  |
|  | del(20q)[1n] |  |
|  | +del(1),+del(11)[1n] |  |
|  | +del(5)[1n] |  |
|  | +del(6),der(13)t(9;13)[1n] |  |
|  | chrb(3),rob(13;13),dup(16),+del(21)[2n] |  |
|  | del(2)(2qter),chtb(4),del(14)(14qter)[2n] |  |
|  | normal karyotype[1n] & [2n][22]* |  |
|  |  |  |
| **1N HAP1 P17[30]** | der(21)t(2;21)[1n] |  |
|  | +del(17)[1n] |  |
|  | chtb(3)[1n] |  |
|  | chtb(19)[1n] |  |
|  | del(3q)[2n] |  |
|  | chrb(10)[2n] |  |
|  | normal karyotype[1n] & [2n][24]* |  |
|  |  |  |
| **HAP1 A1[30]** | del(17)[1n] | +18[1n] |
|  | +del(3)x2[1n] |  |
|  | +del(X)[1n] |  |
|  | +del(2),+2[1n] |  |
|  | +del(13)[1n] |  |
|  | del(10)[2n] |  |
|  | normal karyotype[1n] & [2n][24]* |  |
|  |  |  |
| **HAP1 A2[30]** | chrb(11)[1n] |  |
|  | chtb(18)[1n] |  |
|  | der(8)t(4;8)[1n] |  |
|  | chrb(19)[1n] |  |
|  | der(9)t(5;9;22)[2n] | +4[2n] |
|  |  | +4[2n] |
|  | normal karyotype[1n] & [2n][23]* |  |
|  |  |  |
| **HAP1 A5[30]** | chtb(5),chtb(7)[1n] |  |
|  | der(11)t(2;11)[1n] |  |
|  | del(18)[2n] |  |
|  | normal karyotype[1n] & [2n][27]* |  |
|  |  |  |
| **HAP1 E5[30]** | der[13;(22)t(9;22)],+der[13;(22)t(9;22)]-22[1n] |  |
|  | chrb(2)[1n] |  |
|  | chtb(8),del(13)[1n] | -X[1n] |
|  | +13,+15[1n] |  |
|  | chrb(1)[1n] |  |
|  | chrb(10)[1n] |  |
|  | chrb(1)[1n] |  |
|  | normal karyotype[1n] & [2n][23]* |  |
|  |  |  |
| **HAP1 F3*[30]** | +del(X)[1n] |  |
|  | +del(16)[1n] |  |
|  | chrb(1)[1n] |  |
|  | chrb(2),chrb(10)[1n] |  |
|  | del(1)x2[1n] |  |
|  | der(1)t(1;16),rob(13;13)[2n] | -16[2n] |
|  |  | +22,+22[2n] |
|  | der(2)t(2;10)[2n] | -10,-20[2n] |
|  |  | -13,+22[2n] |
|  | normal karyotype[1n] & [2n][21]* |  |
|  |  |  |
| **HAP1 G2[30]** | rob(13;22)[1n] |  |
|  | chrb(1)[1n] |  |
|  | chrb(7)[1n] |  |
|  | +del(X)[1n] |  |
|  | chrb(4),del(18)[1n] |  |
|  | chrb(10)[1n] |  |
|  | tas(der19;der19)[2n] |  |
|  | chtb(1),+8,+del(10)x3[2n] | -15[2n] |
|  | normal karyotype[1n] & [2n][22]* |  |
|  |  |  |
| **HAP1-HO-C[25]** | der(13)t(3;13)[1n] |  |
|  | der(13)t(13;20p)[1n] |  |
|  | der(13)t(5;13)[1n] |  |
|  | der(8)t(8;12),der(20)t(12;20p),+del(20q)[1n] |  |
|  | normal karyotype[1n] & [2n][26]* |  |
|  |  |  |
| **HAP1 GR 5.10[25]** | der(13)t(8;13)[1n] |  |
|  | der(9)t(9;20;22),der(13)t(9;13)[1n] |  |
|  | der(13)t(9;13)[1n][3]* * |  |
|  | der(13)t[dup(9);13][1n] |  |
|  | i(13;13)[1n] |  |
|  | rob(13;15)[1n] |  |
|  | rob(13;21)[1n] |  |
|  | der(13)t(X;13)[1n] |  |
|  | normal karyotype[1n] & [2n][15]* |  |
|  |  |  |
| **4C1 R1[50]** | +del(3q)[1n] |  |
|  | der(9)t(3;9;22)[1n] |  |
|  | chrb(1)[1n] |  |
|  | der(2)t(2;3),del(16)[2n] |  |
|  | del(3p)[2n] |  |
|  | der(7)t(7;14;17)[2n] |  |
|  | rob(13;13)[2n][2] |  |
|  | del(Xp)[2n] |  |
|  | normal karyotype[1n] & [2n][41]* |  |
|  |  |  |
| **4C1 R2[50]** | +del(3q)[1n] |  |
|  | +del(11p)[1n] |  |
|  | der(4)t(4;15),der(13)t(13;15;22)x2,der(19)t(19;15;19;3)[2n] |  |
|  | del(6q),rob(13;13)[2n] |  |
|  | del(7q)[2n] |  |
|  | der(12)t(12;14)[2n] |  |
|  | del(14)[2n] |  |
|  | chrb(7)[2n] |  |
|  | normal karyotype[1n] & [2n][42]* |  |
|  |  |  |
| **19C1 R1[50]** | 23,X,der(1)t(1;11),der(15)t(1;15)[1n] |  |
|  | 23,X,+del(2p)[1n] |  |
|  | 23,chrb(X)[1n] |  |
|  | 46,X,del(Xq)[2n] |  |
|  | del(5),rob(13;15)[2n] |  |
|  | rob(14;14)[2n] |  |
|  | rob(13;14)x2,+del(14)[2n] |  |
|  | del(13),-ins(19;15)x1[2n] |  |
|  | del(12q)[2n] |  |
|  | normal karyotype[1n] & [2n][41]* |  |
|  |  |  |
| **HAP1 Pool Cas9+Blast[30]** |  | -10,-14[1n] |
|  | der(13)t(9;13)[1n] | -22[1n] |
|  |  | -3[1n] |
|  |  | -17[1n] |
|  |  | -22[1n] |
|  |  | -20[1n] |
|  |  | -18[2n] |
|  | rob(13.13)[2n] |  |
|  |  | -18,-21[2n] |
|  |  | -13,-18[2n] |
|  | del(4)[2n] | -21[2n] |
|  |  | +21[2n] |
|  | +del(5),del(16),del(18)[2n] |  |
|  |  | -15[2n] |
|  | -der(9)t(9;22)x1[2n] |  |
|  | normal karyotype[1n] & [2n][15]* |  |
|  |  |  |
|  |  |  |
|  |  |  |
| **C6 HAP1[30]** | chtb(12)[1n] |  |
|  | normal karyotype[1n] & [2n][29]* |  |

| Novel N- and S-CIN in near-haploid cell lines | |
| --- | --- |
| chrb – chromosome break; chtb- chromatid break; near haploid karyotype - 23,X,t(9;22),ins(19;15); double haploid karyotype - 46,XX,t(9;22)x2,ins(19;15)x2  *metaphases with normal karyotype | |
| **3 metaphases carry the same rearrangement [der(13)t(9;13)] |  |
