# Supplementary material for: Chromosomal instability (CIN) in HAP1 cell lines revealed by multiplex fluorescence in situ hybridisation (M-FISH)
Source: Mol Cytogenet. 2022 Oct 26;15:46. doi: 10.1186/s13039-022-00625-x (PMC9609465; doi:10.1186/s13039-022-00625-x)
Supplement: Supplementary file 2 — Additional file 2: Table S2. Novel N- and S-CIN in double-haploid cell lines. [file 13039_2022_625_MOESM2_ESM.docx]

**Supplementary table 2 (S2)** CIN in double-haploid cell lines

| **Double haploid cell line ID and number of metaphases karyotyped** | **Number of metaphases with novel structural rearrangements (S-CIN); number of metaphases shown in brackets** | **Number of metaphases with novel numerical rearrangements (N-CIN); number of metaphases shown in brackets** |
| --- | --- | --- |
|  |  |  |
| HAP1-P53-KO (30) | del(Xp)[5] |  |
|  |  | -X[6] |
|  | del(Xp)[2] | -22[2] |
|  | del(Xp)[1] | -10,-14[1] |
|  | t(10;17)[1] | -22[1] |
|  |  | -X,-7,-8[1] |
|  | der(X)t(X;13)[1] |  |
|  | der(Xp)t(X;13)[1] | -11[1] |
|  | +del(5)[1] | -8[1] |
|  | del(Xp),del(1q)[2] |  |
|  |  | -X,-14[1] |
|  | der(15)t(X;15)[1] | -X[1] |
|  | del(2)[1] | -X[1] |
|  | der(X)t(X;19),dup(2)?,ins(19;15)x1,del(15)[1] |  |
|  | der(X)t(X;5)[1] |  |
|  |  | -22[1] |
|  | der(3)t(3;15)[1] | -1,-10, -16[1] |
|  | +chrb(1)x1[1] |  |
|  | chrb(2)x1[1] | -X[1] |
|  |  |  |
| HAP1-2N-C (25) | 46,XX[1] |  |
|  | der(1)t(1;12)x2;del(1q);chrb(3)[1] |  |
|  | Iso(1q); Iso(1p);+del(1p)[1] |  |
|  | Iso(1q)[1 deleted],Iso(1p)+1[1] |  |
|  | Iso(1q),Iso(1p),+del(1p)[1] |  |
|  | dup(1q),del(Xp)[1] |  |
|  | Iso(1q)x2; Iso(1p)x2[1] |  |
|  | Iso(1q),Iso(1p)[2] |  |
|  | Iso(1q),Iso(1p),+der(1)t(1;8)[3] |  |
|  | del(Xp)[5] |  |
|  | Iso(1q); Iso(1p);der(13)t(1;13)[4] |  |
|  | rob(13;15)[1] |  |
|  | der(13)t(10;13),chtb(1)[1] |  |
|  |  | -X[2] |

Novel N- and S-CIN in double-haploid cell lines
